# Purposefully Designed Surfactants for Facile and Controllable Gold Colloidal Nanocrystal Synthesis

**DOI:** 10.1021/acsomega.3c05795

**Published:** 2023-10-23

**Authors:** Nakara Bhawawet, Luis Polo-Parada, Piyuni Ishtaweera, Nathaniel E. Larm, Gary A. Baker

**Affiliations:** † Department of Chemistry, Chulalongkorn University, Bangkok 10330, Thailand; ‡ Department of Medical Pharmacology and Physiology, University of Missouri, Columbia, Missouri 65211, United States; § Dalton Cardiovascular Research Center, University of Missouri, Columbia, Missouri 65211, United States; ∥ Department of Chemistry, University of Missouri, Columbia, Missouri 65211, United States; ⊥ Department of Chemistry, United States Naval Academy, Annapolis, Maryland 21402, United States

## Abstract

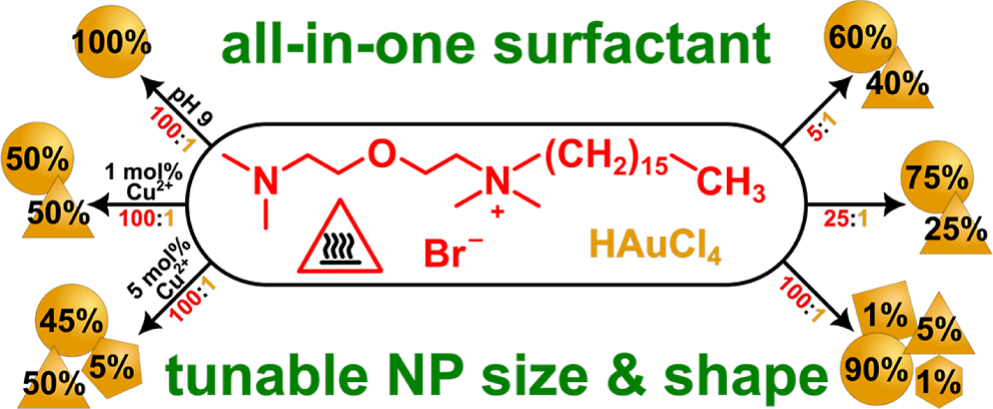

Three new cationic surfactants—*N*-cetyl-bis(2-dimethylaminoethyl)ether bromide (CBDEB), *N-*dodecyl-bis(2-dimethylaminoethyl)ether bromide (DBDEB), and *N*-hexyl-bis(2-dimethylaminoethyl)ether bromide (HBDEB)—have been designed herein using a simple and tailorable synthesis route. CBDEB and DBDEB, the 16- and 12-carbon chain surfactants, demonstrate facile, rapid, and controllable aqueous syntheses of gold nanoparticles (AuNPs) as dual-action reducing and capping agents. The synthesis strategy, using only surfactant and HAuCl_4_ salt, and 4 min of heating at 80 °C, results in spherical AuNPs (average diameters of 13.4 ± 3.8 nm for CBDEB and 12.0 ± 3.8 nm for DBDEB). Microwave irradiation was also investigated as a heating method and produces AuNPs in as little as 30 s. Control over the size and shape of AuNPs was proven to be feasible (toward populations of Euclidean shapes) by appropriately tuning reaction parameters, such as the molar ratio of surfactant to Au^3+^, temperature, incorporation of a time delay before heating, or shape control agents, such as Cu^2+^. Frustratingly, the cytotoxicity of CBDEB is similar to that of cetyltrimethylammonium bromide (CTAB), a popular 16-carbon chain cationic surfactant. Notably, while the shorter HBDEB (6-carbon chain) does not produce AuNPs under the applied conditions, it does appear to improve cell viability upon cytotoxicity evaluation and may be favorable as a new biological surfactant.

## 1 Introduction

Traditional surfactants are amphiphiles with hydrophilic head groups and lipophilic tails. In aqueous media, these molecules self-associate to form dynamic assemblies known as micelles, colloids wherein the polar head groups interact with the medium while the nonpolar tails are sequestered within the micellar interior.^1^ The minimum concentration at which these micelles spontaneously form is known as the critical micelle concentration (CMC) and is a crucial parameter when using surfactants for solubilization and dispersion.^2,3^ More recently, the study of micelles and surfactants has extended beyond analyte dissolution, extraction, and transport toward “nano-reactor” (i.e., chemistry in a micelle) and controlled/constrained nanoparticle (NP) synthesis applications.^1,4,5^

In nanochemistry, the term “stabilizing ligand” refers to any molecule that binds to the surface of a metal NP to control its growth and colloidal stability (dispersibility, surface charge, tendency to aggregate). Often, these stabilizing ligands are referred to as “surfactants” due to their role in lowering the liquid–solid interfacial tension between the medium and the NP. These interchangeable terms lead to indistinct and confusing verbiage since some traditional micelle-compliant surfactants lack the characteristic NP interactions of stabilizing ligands, while some traditional stabilizing ligands do not possess the distinguishing micelle compliance of surfactants (e.g., citrate, ascorbate, and borohydride lack the ability to form a micelle in aqueous media). The intersection of these two roles creates an interesting frontier in colloid chemistry where surfactant-induced micelles sequester metal atoms and template the formation of NPs, followed by a role switch where the surfactant acts as a stabilizing ligand and controls the interfacial chemistry between the formed NPs to prevent aggregation. A few common examples of the popular surfactant/stabilizing ligand molecules are oleylamine and dodecanthiol.^6−9^ The NPs prepared by using these surfactants exhibit excellent monodispersity and colloidal stability, making their colloids ideal for applications that exploit their plasmonic properties. Indeed, the search for similar amphiphiles is a constant undertaking and can lead to the design of novel surfactants with wide-reaching uses.

Herein, we purposefully designed *N*-cetyl-bis(2-dimethylaminoethyl)ether bromide (CBDEB), a new surfactant, reducing agent, and stabilizing ligand for facile and controllable gold NP (AuNP) synthesis. Two shorter alkyl analogues, *N*-dodecyl-bis(2-dimethylaminoethyl)ether bromide (DBDEB) and *N*-hexyl-bis(2-dimethylaminoethyl)ether bromide (HBDEB), were also prepared to elucidate the correlations between alkyl chain length and surfactant cytotoxicity, average AuNP size, and AuNP shape and monodispersity.

## 2 Experimental Section

### 2.1 Materials

All experiments were carried out using ultrapure Millipore water polished to a purity of 18.2 MΩ·cm. Bis[2-(*N*,*N*-dimethylamino)ethyl] ether (667,609, 97%), 1-bromohexadecane (234451, 97%), 1-bromododecane (B65551, 97%), hexadecyltrimethylammonium bromide (CTAB, H5882, 98%), and tetrachloroauric acid trihydrate (520918, ≥99.9% trace metal basis) were all purchased from Sigma-Aldrich (St. Louis, MO). Toluene (T290SK, HPLC) and anhydrous ethyl ether (E138, Certified ACS) were purchased from Fisher Scientific (Hampton, NH).

### 2.2 Materials, Synthesis, and Procedures

#### 2.2.1 Synthesis of the Surfactants (CBDEB, DBDEB, and HBDEB)

**Scheme 1:**
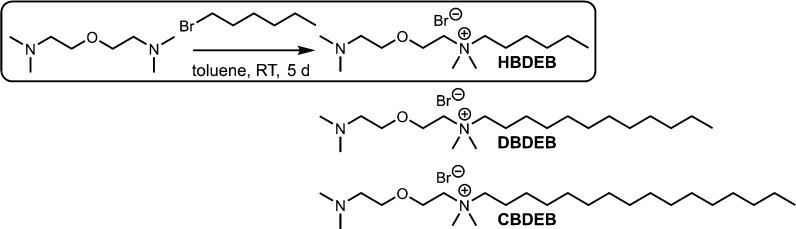
Reaction Diagram Describing Surfactant Preparation and Reaction Conditions The alkyl chain length in the product can be tailored by changing the identity of the bromoalkane precursor.

An alkylation reaction was performed in a round-bottom flask equipped with a magnetic stir bar. Approximately 10 g of bis[2-(*N*,*N*-dimethylamino)ethyl] ether was dissolved in 100 mL of toluene, followed by the slow addition of an equimolar quantity of 1-bromoalkane (alkyl chain length varied based on the desired product) (Scheme 1). The reaction mixture was allowed to stir for 5 days at room temperature, resulting in generation of the product as a white precipitate. The product was centrifuged and washed with toluene three times to remove the unreacted starting materials. The crude solid was then filtered and washed again with diethyl ether and then dried under vacuum.

#### 2.2.2 Cell Viability Using the Calcein-AM Assay

SK-MEL-28 cells, sourced from ATCC (Manassas, VA), are melanocytes isolated from the skin tissue of a 51 year old male patient with malignant melanoma. The viability of SK-MEL-28 cells treated with HBDEB, DBDEB, and CBDEB quaternary ammonium salts was determined by a calcein-AM assay using cells seeded within 96-well plates. After 24 h of incubation at different quat concentrations, cell viability was quantitated using calcein-AM assay using modifications to a previously described protocol.^10^ This cytosol-enzyme-specific live cell labeling assay is based on measuring the green fluorescence content of surviving cells as an index to determine cell growth, inhibition, and viability. In brief, 5 to 8 × 10^3^ SK-MEL-28 cells in 100 μL of culture media were seeded into each well of a 96-well plate and incubated overnight at 37 °C under 10% CO_2_. The medium was removed, and cells were washed with 100 μL of serum-free Dulbecco’s modified Eagle medium (DMEM/F12) once and then treated in a 10% fetal bovine serum culture medium for 24 h in the presence of 0.1 to 30 μL of stock solutions of the HBDEB, DBDEB, and CBDEB salts using the common shape-directing quaternary ammonium surfactant cetyltrimethylammonium bromide (CTAB) as a benchmark for comparison. After treatment, the medium was removed, cells were washed with PBS, and surviving adherent cells were labeled by incubating the cells in the same culture medium containing calcein-AM (5 μM) at 37 °C for 30 min. The cells were washed with the culture medium to remove the excess probe, and then the fluorescence intensity was determined on a fluorescence plate reader (Promega, GloMax Explorer multimode microplate reader) using 490 nm excitation while monitoring 525 nm emission. Three wells were made per sample condition, and each sample was measured in triplicate.

#### 2.2.3 Synthesis of the AuNPs

In a typical synthesis using the conventional heating method, 20 μL of 100 mM aqueous HAuCl_4_ was added to a two-dram vial containing 1.00 mL of a surfactant (e.g., 100 mM aqueous surfactant for the 50:1 molar ratio of surfactant to Au), which was preheated at 80 °C and stirred on a hot plate equipped with a reaction block. For the microwave heating method, 20 μL of 100 mM aqueous HAuCl_4_ was placed in a glass microwave vial containing 1.00 mL of a surfactant. A commercial research-grade microwave reactor (CEM Corporation, Discover SP, Model no. 909150) was set to prestir the solution for 30 s prior to ramping the temperature at an approximate rate of 1.07 °C s^–1^ to a designated final temperature (40–100 °C), which was then maintained for 15–300 s. Once the reaction was completed (for either heating method), the products were isolated by centrifugation at 15,000 rpm for 15 min and the supernatants were carefully removed. A 1.00 mL aliquot of water was then added to the product to redisperse the NPs by gentle shaking. This washing process was repeated two more times and the final products were redispersed in 1.00 mL of water.

### 2.3 Characterization

A Bruker AVIII 500 MHz NMR spectrometer was used to characterize the surfactants. Mass spectroscopy (MS) data were collected in positive-ion QTOF-MS mode from 20 to 1300 *m*/*z* (timsTOF Pro 2; Bruker Scientific Instruments, Billerica, MA). NanoESI-positive data (1.6 kV cap voltage) were collected by infusing methanolic compound at 0.5 μL min^–1^ from a 500 μL Hamilton syringe. Data were acquired over 1 min of infusion. The MS was calibrated with ESI-low (Agilent) just prior to acquisition. All UV–vis spectra were recorded on a Cary 60 UV–vis spectrophotometer using 1 cm path length disposable poly(methyl methacrylate) (PMMA) cuvettes. All samples for UV–vis measurements were 12× diluted and blank subtracted. Transmission electron microscopy (TEM) studies were conducted on carbon-coated copper grids (Ted Pella, Inc. 01814 F, support films, carbon type-B, 400 mesh copper grid) using a FEI Tecnai (F20 G2, Twin) microscope operated at a 200 keV accelerating electron voltage. The CMC was determined by electrical conductivity measurements, which were performed using a conductivity probe equipped with LabQuest Mini interface and Logger Lite software (Vernier Software & Technology). Before the measurements, a two-point calibration was performed using a standard solution with known conductivities (Flinn Scientific). The probe was immersed in stirred sample solutions at ambient temperature (21.8 °C) until a reading was stabilized.

## 3 Results and Discussion

### 3.1 Structure and Characterization of the Surfactants

**Figure 1:**
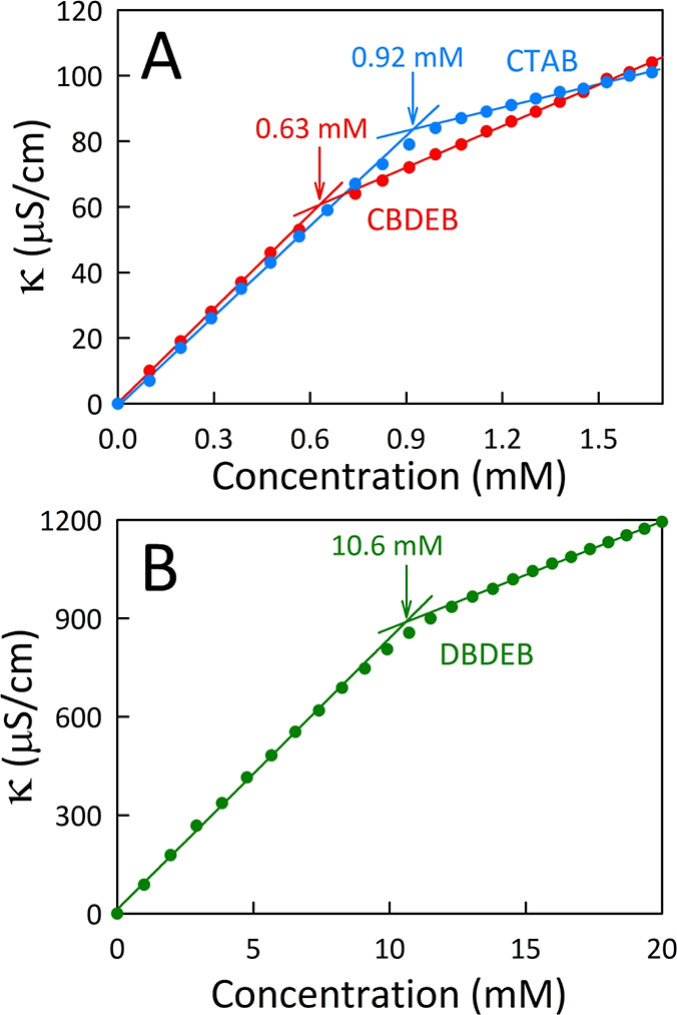
Plots of the specific conductivity of (A) CTAB and CBDEB and (B) DBDEB as functions of concentration in water at 21.8 °C. The solid curves denote linear best fits, and the estimated CMC values are indicated by arrows at their intersections.

The surfactant structures and ^1^H NMR spectra are presented in Figures S1–S3. For illustration, the quadrupole time-of-flight (QTOF) mass spectrum measured in the positive-ion mode for HBDEB is also shown in Figure S4. We report a less than 15 ppm error between the experimental and theoretical mass values for all isotopic distributions observed in the mass spectrum of HBDEB. The clean ^1^H NMR spectra for all compounds and mass spectrometric confirmation corroborate the successful preparation and positive identification of the synthetic surfactants. These surfactants were designed to incorporate a tertiary amine and provide amphiphilicity alongside enough reducing power to convert Au^3+^ to Au^0^ with brief conventional (i.e., hot plate) or microwave (MW) heating (Scheme 1). The CMCs were determined by electrical conductivity titrations; based on the Onsager theory of electrolyte conductivity,^3^ two linear regimes for specific conductivity (κ) are expected above and below the CMC such that the corresponding first derivative profile behaves as a Boltzmann-type reverse sigmoid.^11^ The integration of this function provides an analytical expression that describes the concentration dependence of κ during micellization. We determined that the CMCs of our surfactants CBDEB and DBDEB are 0.63 and 10.6 mM, respectively (Figure 1), and note that HBDEB did not exhibit a distinct CMC. Note that the systematic decrease in CMC with longer alkyl chain length is a common trend for conventional ionic surfactants.^1,12−17^ Interestingly, the CBDEB surfactant has a lower CMC than CTAB, a common 16-carbon chain surfactant. This is likely due to increased hydrophobicity by the dimethylaminoethyl ether headgroup, as also observed for other cationic headgroup surfactants.^18,19^

**Figure 2:**
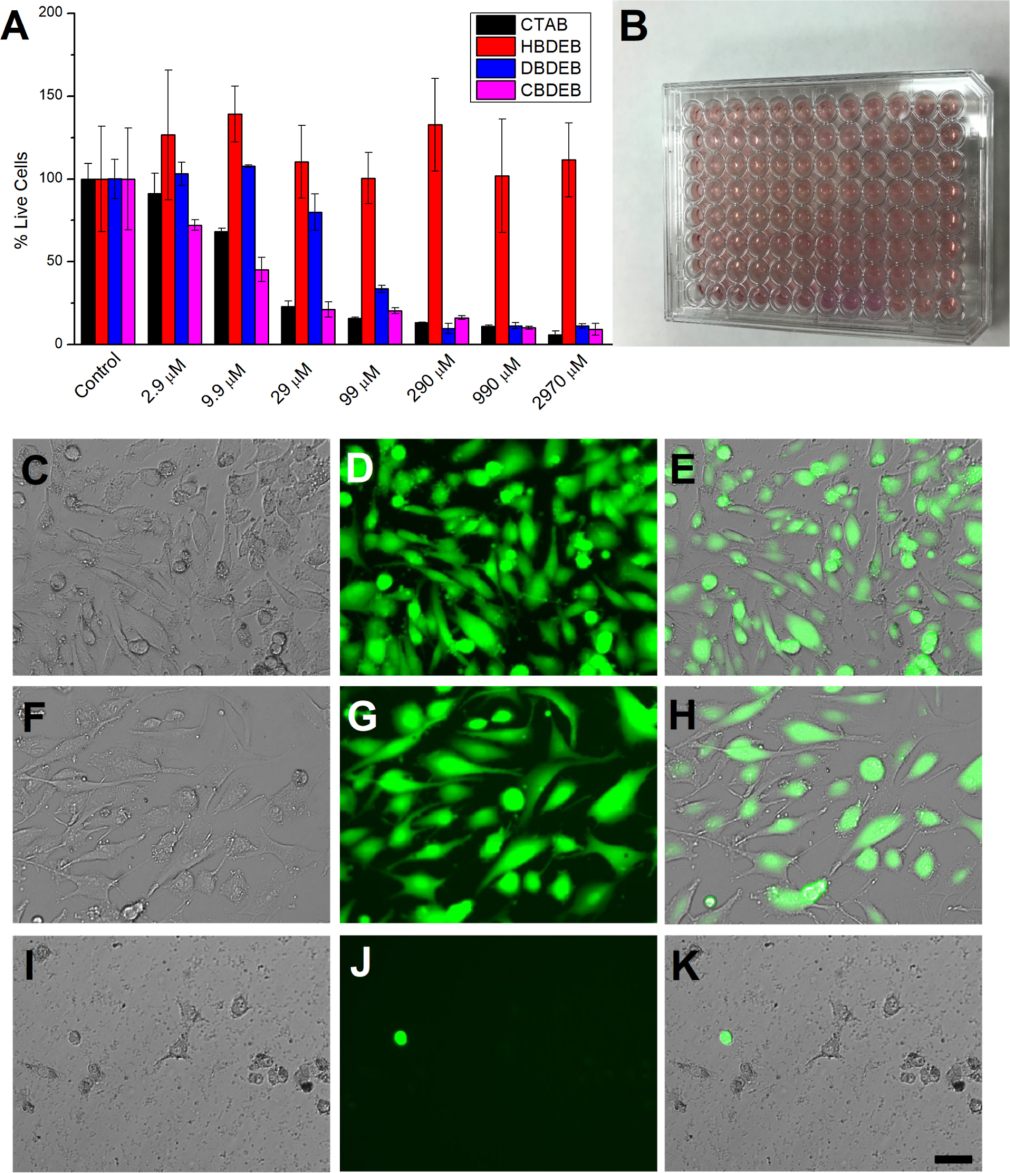
(A) Cell viability of human SK-MEL-28 cells determined from the calcein-AM assay after 24 h exposure to different concentrations of the quaternary ammonium salts HBDEB, DBDEB, and CBDEB (6-, 12-, and 16-carbon aliphatic chain lengths, respectively) using a (B) 96-well microplate reader format. Data represent mean ± SD. (C,F,I) Transmitted light, (D,G,J) fluorescence, and (E,H,K) superposition of transmission/fluorescence images for SK-MEL-28 cells exposed to (C–E) HBDEB, (F–H) DBDEB, and (I–K) CBDEB, each at a 29 μM concentration. Scale bar is 25 μm.

We also assessed the cytotoxicity of the newly synthesized salts using calcein-AM assay, which is a vital dye method for quantifying cell viability. SK-MEL-28 cells (human malignant melanoma cells) were incubated with various concentrations of HBDEB, DBDEB, and CBDEB for 24 h, after which the cytotoxicity was quantified by measuring the resulting green fluorescence at 525 nm using a 96-well microplate reader (Figure 2). In this assay, the acetoxymethyl ester of calcein that passively crosses the cell membrane of viable cells is converted by cytosolic esterases to calcein, whose green fluorescence is retained by cells having intact membranes. We note that the light orange color observed in the plate wells in Figure 2B arises from phenol red (PR). While the presence of PR is not critical for maintaining cell cultures, this standard pH indicator dye has been used for several decades to provide a facile means for researchers to assess the health of various cell and tissue culture media.

The calcein-AM viability assay for the short-chain HBDEB (Figure 2A) showed no dose-dependent effects on the viability of human SK-MEL-28 cells, indicating that the adherent cells were unaffected by this compound at any of the employed concentrations. In fact, the mean cell viability of SK-MEL-28 cells exposed to the hexyl-pendant HBDEB was higher than that of the untreated control cells, suggesting that HBDEB is nontoxic and might offer a safe capping agent alternative for future biological applications. In fact, we observed that the addition of HBDEB appears to promote cell growth by as much as 50% or more in additional cell lines as well, including HeLa (human cervical cancer), PC-12 (adrenal gland pheochromocytoma from rat), HSN36 (human melanoma), B16F10 (mouse melanoma), and PC3-PIP (human prostate) cells (data not shown). HBDEB possesses a chain length near that of short-chain fatty acids (SCFAs) which are easily metabolized by many cells. Indeed, SCFAs can be broken down by β-oxidation to generate acetyl-CoA units which can enter into the TCA cycle where they are oxidized for energy production. Notably, increased levels of SCFAs and acetyl-CoA appear to promote cell activation and antibody production. As a carrier of acyl groups, acetyl-CoA is also an essential cofactor in post-translational acetylation reactions. Thus, if HBDEB is processed by a similar route to SCFAs, this would explain our observations. Ongoing experiments seek to elucidate this hypothesis but are beyond the scope of the current work.

The aliphatic chain length of the amine-terminated quaternary ammonium salt (*n* = 6, 12, or 16) played a critical role in the extent of SK-MEL-28 cell mortality. While no significant toxicity was observed for the lowest concentrations of DBDEB, this salt was highly cytotoxic to adherent SK-MEL-28 cells when present at 99 μM or more. For better context, the dose-dependent toxicity of CTAB is provided alongside in Figure 2A. CTAB is a popular growth-directing surfactant employed for shape control during nanoscale synthesis and is associated with marked cytotoxicity,^20^ an effect variously attributed to free CTAB in solution (e.g., dissociation from the NP surface)^21^ or the unique bilayer structure of the CTAB molecules on NP surfaces.^22^ The toxicity of CTAB is notably highly dependent on concentration and cell type and resulted in a significant reduction in the integrity of lysosomal membranes of A549 cells (lung carcinoma epithelial cells) at a concentration of only 1 μM.^23^ The results in Figure 2A reveal that the same aliphatic-chain length analogue CBDEB is essentially as cytotoxic as CTAB, something we might have anticipated from the presence of the hexadecyl chain in both. Indeed, as can be seen in panel J of Figure 2, the lack of green fluorescence demonstrates extremely low cell viability, even at a low concentration of 29 μM for CBDEB.

### 3.2 Synthesis of AuNPs

**Figure 3:**
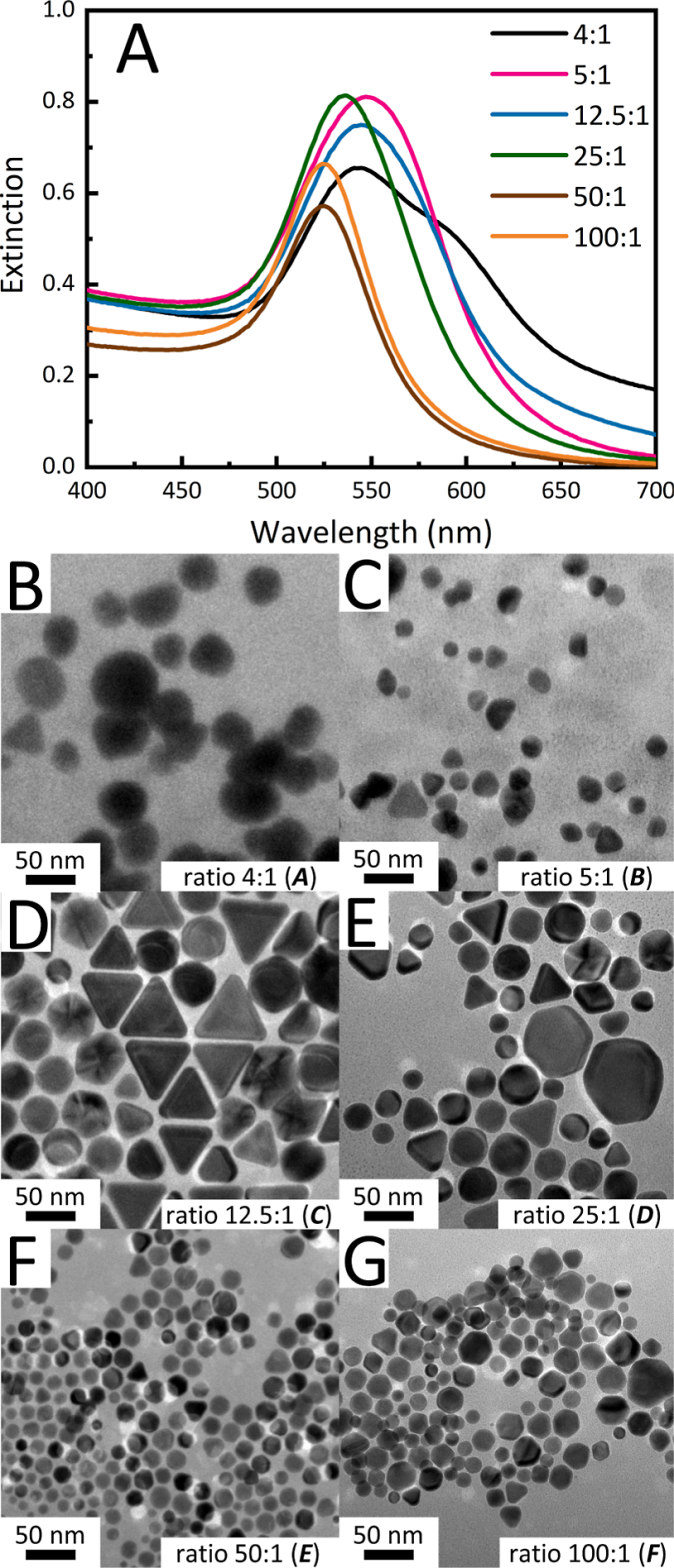
(A) UV–vis spectra comparing AuNP dispersion synthesized from various CBDEB to Au^3+^ molar ratios. (B–G) Representative TEM images of AuNPs synthesized using the CBDEB to Au^3+^ molar ratios of 4:1, 5:1, 12.5:1, 25:1, 50:1, and 100:1, respectively.

The CBDEB surfactant was investigated for the synthesis of AuNPs, as amines have been reported as reducing and stabilizing agents for AuNPs.^24^ For this investigation, a 20 μL aliquot of 100 mM aqueous HAuCl_4_ was rapidly added to 1.00 mL of aqueous CBDEB (concentration varied) maintained at 80 °C on a heating block. The solution was stirred for 10 min before the assessment of AuNP formation. A 3:1 molar ratio of surfactant to Au^3+^ resulted in a hazy yellow solution, indicative of failed AuNP formation (typical AuNP colloids appear wine red to purple due to their plasmonic properties). The reactions of the 4:1 molar ratio (designated as sample A; this nomenclature is used throughout to match NP colloids with their entries in Table S1) and 5:1 molar ratio (B) resulted in purple suspensions, while the molar ratios of 12.5:1 (C), 25:1 (D), 50:1 (E), and 100:1 (F) produced purple, magenta, ruby red, and ruby red colloids, respectively (Figure S5). UV–vis absorption spectrophotometry was used as a screening tool to predict the size and distribution of the synthesized AuNPs. The resulting spectra, provided in Figure 3A, reveal that the localized surface plasmon band (LSPR) narrows and shifts toward the blue as the molar ratio of the surfactant increases, with ratios of 50:1 and 100:1 (E and F, respectively) expressing nearly identical plasmon bands. To elucidate the temporal element of AuNP formation, a 50:1 molar ratio CBDEB-Au^3+^ sample was heated for 15 min while aliquots were taken at several time intervals and the LSPR intensity was monitored at 522 nm (Figure S6). Interestingly, a reaction time of 4 min achieves maximal development of the plasmon band after which the extinction plateaus.

In light of this spectral trend and the general colloid appearances, we initially expected that larger and more polydisperse spherical AuNPs were obtained for lower ratios of surfactant to Au^3+^, while higher ratios favored smaller and more monodispersed spherical AuNPs. Surprisingly, TEM analysis reveals a wide variety of AuNP morphologies, namely, circular, triangular, hexagonal, pentagonal, rod-like, and square. While the circular (likely quasispherical) shape is the most abundant, triangular AuNP plates are present in substantial amounts in all the samples. In Table S1, we provide an approximate distribution (%) for each observed shape and the average sizes of the spherical and triangular AuNPs. The data in Table S1 suggest that, in general, lower surfactant to Au^3+^ ratios result in a larger population of triangular AuNPs, while higher molar ratios yield more spherical AuNPs. In Figure 3B–G, we provide representative TEM images of the AuNPs generated using each of the ratios studied. It was found that the ratio of 12.5:1 (sample C) produced the highest quantity of triangular AuNPs (41.2%) and the ratio of 50:1 (E) produced the highest quantity of spherical AuNPs (93.7%) with an average particle size and distribution of 13.4 ± 3.8 nm. While the ratios of 50:1 (sample E) and 100:1 (sample F) expressed similar spectral shapes, the 50:1 ratio produced smaller and more uniform AuNPs; therefore, the 50:1 surfactant to Au^3+^ ratio was selected as the optimum synthesis parameter for further studies.

A sample solution was prepared without heating to establish whether thermal input is necessary for reduction to occur. A 20 μL aliquot of 100 mM aqueous HAuCl_4_ was added to 1.00 mL of the CBDEB surfactant (50:1 molar ratio of CBDEB to Au) at room temperature and stirred for 0–48 h. The resulting spectra indicate that Au^3+^ is fully reduced by the surfactant at ambient conditions within 6 h, after which the intensity of the extinction peak stays relatively constant and the overall spectral shape narrows (Figure S7). The AuNPs generated after 6 h (G) exhibited agglomeration and a primarily triangular (41.3%) and spherical (64.2%) population (Table S1 and Figure S8).

**Figure 4:**
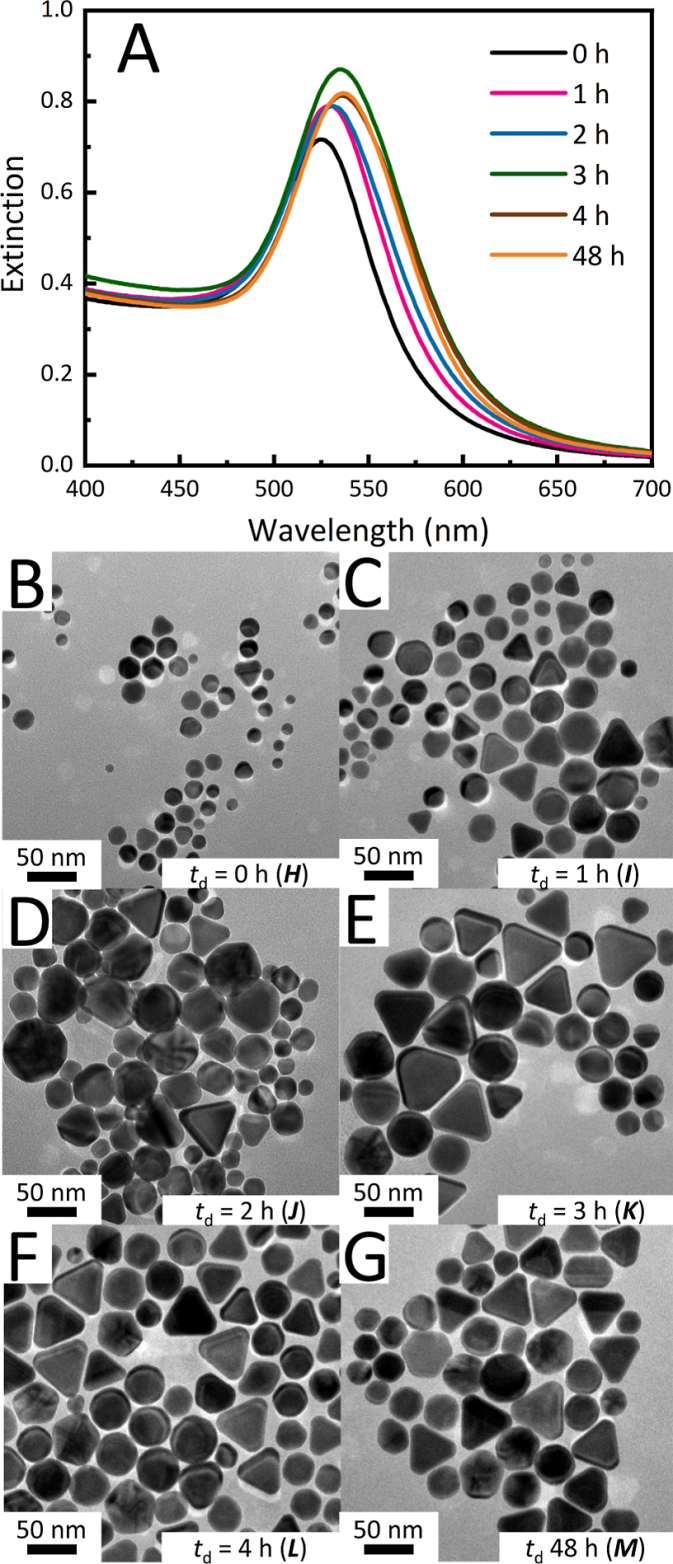
(A) UV–vis spectra of AuNPs showing bathochromic shifts with longer delayed time before heating from 0 to 3 h. After 3 h, the spectral position remained unchanged up to 48 h. (B–G) Representative TEM images of AuNPs generated with heating after a set delay time of 0 h (sample H), 1 h (I), 2 h (J), 3 h (K), 4 h (L), and 48 h (M) h.

Heating profiles were introduced to tune the AuNP formation and distribution with a delay time (*t*_d_) between adding HAuCl_4_ to the surfactant solution and the start of heating. Using consistent reaction compositions (similar to previous experiments with a 50:1 molar ratio of CBDEB to Au), the resulting solution was stirred at room temperature for a designated *t*_d_ before being placed in a preheated aluminum block set to 80 °C for 4 min. The incorporation of a preheat delay causes red-shifting and peak broadening in the prepared AuNPs, as measured by the full peak width at 75% (FW@0.75 max) of the LSPR band, with more pronounced effects observed as *t*_d_ approaches 3 h (Figure 4A and S9). A *t*_d_ greater than 3 h did not noticeably influence the LSPR band. TEM analysis indicates that the population of spherical AuNPs decreases from 88.5 to 52.2% (while the population of triangular shapes increases from 10.0 to 41.3%) as the *t*_d_ increases from 0 to 48 h (samples H–M, Table S1). We provide the representative TEM images for these samples in Figure 4B–G.

Microwave irradiation is a common heating source for NP production and is known to uniformly heat and shorten reaction times.^25^ In brief, 20 μL of 100 mM aqueous HAuCl_4_ was transferred to a microwave vial containing 1.00 mL of CBDEB surfactant. The CBDEB concentration, maximal temperature, and hold time (*t*_h_) at max temperature were optimized to produce a colloid with the most blue-shifted extinction profile and narrowest FW@0.75 max (Table S2 and Figure S10). The determined optimal conditions, a 50:1 molar ratio of CBDEB/Au and a max temperature of 60 °C, a *t*_h_ of 30 s, yield a ruby red colloid (sample N) with an LSPR peak at 525 nm (Figure S11, red) which is indistinguishable from the optimal spectrum generated with conventional heating (sample E; Figure S11, blue). TEM analysis reveals a slightly larger spherical particle size (14.7 ± 4.5 nm) as well as a broader particle size distribution (Figure S12).

### 3.3 Tailoring the AuNP Size and Morphology

Surfactant chain length was briefly investigated as a variable of AuNP formation. DBDEB and HBDEB (12 and 6 carbon chains, respectively) were separately applied to the conventional heating method using a 50:1 molar ratio of surfactant to Au. For DBDEB, the resulting colloid (O) appeared ruby red and expressed a slightly broader LSPR band (Figure S11, green) as compared to colloid E (CBDEB). TEM images of AuNPs from colloid O depict slightly smaller spherical particles (12.0 ± 3.8 nm; Figure S13). Unfortunately, HBDEB did not produce AuNPs under these conditions. In fact, a side experiment employing sodium borohydride (NaBH_4_) as the primary reducing agent in the presence of HBDEB also failed to produce stable NPs from the reduction of gold salt and instead produced unstable colloids that quickly settled from solution as a fine black precipitate (room temperature solution comprising 2 mM HAuCl_4_ for a BH_4_/Au/HBDEB molar ratio of 10:1:50). This result points to the importance of ligand amphiphilicity in producing stable colloidal NPs. Control experiments, substituting CTAB or NaBr for the surfactant, were conducted to ensure that they could not individually reduce Au^3+^ to form AuNPs without the tertiary amine present in our surfactants. Conventional heating procedures were used, and neither substitution resulted in AuNP formation (Figure S14). Accordingly, 2-(dimethylamino)ethyl ether (DMAEE; surfactant precursor) possesses two tertiary amines and reduces Au^3+^ to create blue and highly aggregated AuNPs (Figure S15). Similar results were observed when DMAEE and CTAB were used in the microwave method (Figure S15). These controls confirm the crucial design elements for trifunctional micelle-forming/reducing/stabilizing surfactants.

In addition, alkalinity has been reported to have an effect on the morphology of the AuNPs.^26,27^ To examine the effects of acid and alkaline conditions on the formation of AuNPs, 25–100 μL of 1 M HCl or 1 M NaOH was added to the 80 °C preheated surfactant solution (originally pH 9) prior to the addition of HAuCl_4_. Notably, increasing acidity noticeably slowed the AuNP formation (ca. 7 min to achieve a ruby red solution after addition of 75 μL of 1 M HCl; Figure S16A). Conversely, alkaline reactions were significantly more rapid [e.g., nearly instantaneous color change after adding 75 μL of 1 M NaOH (sample *P*); Figure S16B]. The LSPR bands of the alkaline colloids are also blue-shifted and broader than those produced under acidic conditions, suggesting polydisperse particles. TEM analysis of colloid *P*, being selected as a representative sample, confirms the polydispersity and high degree of agglomeration of the AuNPs (Figure S17).

Finally, we investigated the addition of CuSO_4_ to selectively prepare triangular AuNPs, as the shape control effects of Cu^2+^ have been shown in a report from Takezaki et al.^28^ Briefly, 1, 5, and 10 mol % CuSO_4_ relative to Au^3+^ (colloids Q, R, and S, respectively) were added into the preheated surfactant solutions before the addition of HAuCl_4_, with the remainder of the synthesis being performed as described in our optimal protocol. The resulting UV–vis spectra (Figure S18A) show a monotonic red shift of the LSPR band as the relative concentration of CuSO_4_ increases. TEM analysis (Table S1 and Figure S18B–G) reveals that an increase in Cu^2+^ concentration also increases the population of triangular shapes and overall AuNP size; in the presence of 10 mol % Cu^2+^ to Au (sample S), triangular AuNPs dominate the population (54.2%) with average side length of 62.8 ± 9.3 nm. Conversely, samples comprising 5 (R) and 1 (Q) mol % Cu^2+^ exhibit 51.3 and 45.7% triangular AuNP populations with average side lengths of 60.3 ± 9.1 and 46.3 ± 8.6 nm, respectively.

## 4 Conclusions

In summary, a new cationic surfactant with a tertiary amine, *N*-cetyl-bis(2-dimethylaminoethyl)ether bromide (CBDEB, 16-carbon chain), was purposefully prepared for the facile and rapid synthesis of AuNPs. Two related surfactants with shorter aliphatic chains—*N*-dodecyl-bis(2-dimethylaminoethyl)ether bromide (DBDEB, 12-carbon chain) and *N*-hexyl-bis(2-dimethylaminoethyl)ether bromide (HBDEB, 6-carbon chain)—were also prepared, demonstrating the synthesis of surfactants with tailorable aliphaticity. The CMCs of CBDEB and DBDEB were determined to be 0.63 and 10.6 mM, respectively (indeterminate CMC for HBDEB), and CBDEB was found to sharply reduce the viability of human SK-MEL-28 cells as determined from calcein-AM assay (comparable cytotoxicity to CTAB, a common 16-carbon chain cationic surfactant). Interestingly, HBDEB is demonstrably noncytotoxic and may, in fact, actually promote cell growth. This discovery is alluring when considering the simple synthesis method for HBDEB, and tailoring product aliphaticity to create an assortment of noncytotoxic surfactants is an interesting prospect. CBDEB, a dual-function reducing/stabilizing agent for the simple and rapid aqueous synthesis of HAuCl_4_, produces a red aqueous colloid of uniform AuNPs with a predominantly spherical shape (average size of 13.4 ± 3.8 nm). This occurs within minutes when heating an aqueous solution of surfactant and gold salt precursor (surfactant to Au^3+^ molar ratio of 50:1) at 80 °C. The product can be tuned toward triangular shapes by (1) decreasing the molar ratio of surfactant to Au^3+^, (2) delaying the application of heat, or (3) adding CuSO_4_ as a shape-directing agent. Under similar conditions, DBDEB also acts as a dual-function surfactant (achieving an average spherical AuNP size of 12.0 ± 3.8 nm), while a solution containing HBDEB did not seem to produce AuNPs. Overall, CBDEB and DBDEB prove to be interesting reductants and stabilizing ligands for AuNP formation, whereas HBDEB may be a safe capping agent for biological systems.

## Supporting Information Available

Tabulated AuNP sizes and distributions, sample photographs, ^1^H NMR spectra, UV–vis spectral analysis of colloids, and TEM images of samples (PDF)
