# Supplementary material for: Purposefully Designed Surfactants for Facile and Controllable Gold Colloidal Nanocrystal Synthesis
Source: ACS Omega. 2023 Oct 23;8(44):41633–40. doi: 10.1021/acsomega.3c05795 (PMC10633875; doi:10.1021/acsomega.3c05795)
Supplement: Supplementary file 1 — ao3c05795_si_001.pdf [file ao3c05795_si_001.pdf]

SUPPORTING INFORMATION

Purposefully Designed Surfactants for Facile and Controllable Gold Colloidal Nanocrystal Synthesis

Nakara Bhawawet[1], Luis Polo-Parada[2,3], Piyuni Ishtaweera[4], and Nathaniel E. Larm[5,*], Gary A. Baker[4,*]

[1] Department of Chemistry, Chulalongkorn University, Bangkok 10330, Thailand
[2] Department of Medical Pharmacology and Physiology, University of Missouri, Columbia, MO 65211, USA.
[3] Dalton Cardiovascular Research Center, University of Missouri, Columbia, MO 65211, USA.
[4] Department of Chemistry, University of Missouri, Columbia, MO 65211, USA.
[5] Department of Chemistry, United States Naval Academy, Annapolis, MD 21402, USA

*Email: larm@usna.edu, bakergar@missouri.edu

Table S1. Approximate percentage shape distribution of AuNPs and average size of spherical and triangular particles.

sample	round	triangular	hexagonal	pentagonal	rod	square/ rhombus
**% shape distribution**						
**average size (nm)**						
Surfactant: Au^{3+}	20 μL of 100 mM $HAuCl_4$ was added to a pre-heated solution at 80 °C of CBDEB					
4:1 (***A***)	*Unidentifiable due to massive cold welding*					
	49.4 ± 16.1					
5:1 (***B***)	58.8	40.7	0.0	0.0	0.5	0.0
	19.7 ± 4.3	31.2 ± 8.0				
12.5:1 (***C***)	49.2	41.2	4.8	3.7	0.8	0.0
	26.7 ± 9.0	53.3 ± 13.6				
25:1 (***D***)	74.1	22.2	1.1	0.6	0.8	0.9
	23.0 ± 7.8	42.5 ± 12.4				
50:1 (***E***)	93.7	4.2	2.0	0.0	0.0	0.0
	13.4 ± 3.8	19.0 ± 3.4				
100:1 (***F***)	89.8	3.8	4.9	0.2	0.2	1.1
	15.7 ± 5.1	31.6 ± 10.5				
(***G***)	20 μL of 100 mM $HAuCl_4$ was added to 100 mM CBDEB at room temperature. The reaction was stopped at 6 h without heating.					
	64.2	31.7	1.6	1.6	0.8	0.1
	29.5 ± 11.4	54.5 ± 18.4				
	20 μL of 100 mM $HAuCl_4$ was added to 1.00 mL of 100 mM CBDEB at room temperature. After set delayed time, the sample was brought to heating at 80 °C and held for 4 min.					
delayed 0 h (***H***)	88.5	10.0	1.2	0.0	0.3	0.0
	14.9 ± 3.8	22.0 ± 4.4				
delayed 1 h (***I***)	83.1	15.0	0.5	0.4	0.3	0.4
	20.4 ± 8.5	36.6 ± 10.4				
delayed 2 h (***J***)	81.4	13.5	0.6	0.1	2.0	1.6
	22.3 ± 10.1	37.7 ± 15.3				
delayed 3 h (***K***)	69.3	26.8	0.9	1.2	1.0	0.3
	30.8 ± 10.8	52.5 ± 13.8				
delayed 4 h (***L***)	64.2	31.7	1.6	1.6	0.8	0.1
	29.0 ± 10.8	49.7 ± 12.9				
delayed 48 h (***M***)	52.2	41.3	4.3	1.4	0.7	0.0
	31.5 ± 9.8	48.4 ± 10.5				

Table S1 (continued). Approximate percentage shape distribution of AuNPs and average size of spherical and triangular particles.

Sample	Round	Triangular	Hexagonal	Pentagonal	Rod-like	Square/ Rhombus
**% Shape distribution**						
**Size (nm)**						
Microwave irradiation (***N***)	20 μL of 100 mM $HAuCl_4$ was added to 1.00 mL of 100 mM CBDEB. The mixture was microwave heated at 60 °C for 30 s.					
	91.5	7.6	0.6	0.1	0.0	0.0
	14.7 ± 4.5	22.1 ± 4.3				
Shorter chain surfactant DBDEB (***O***)	20 μL of 100 mM $HAuCl_4$ was added to a pre-heated (80 °C) solution of 1.00 mL of 100 mM DBDEB					
	90.8	7.0	1.1	0.5	0.3	0.3
	12.0 ± 3.8	14.5 ± 3.8				
Alkaline condition (***P***)	20 μL of 100 mM $HAuCl_4$ was added to a pre-heated at 80 °C a solution mixture of 1.00 mL of 100 mM CBDEB and 75 μL of 1 M NaOH					
	14.6 ± 11.0					
	20 μL of 100 mM $HAuCl_4$ was added to a pre-heated (80 °C) solution of 1.00 mL of 100 mM CBDEB and $CuSO_4$					
1 mol% Cu^{2+} (***Q***)	49.8	45.7	1.0	2.5	0.9	0.1
	31.8 ± 7.8	46.3 ± 8.6				
5 mol% Cu^{2+} (***R***)	42.5	51.3	0.4	4.0	1.5	0.4
	37.2 ± 12.2	60.3 ± 9.1				
10 mol% Cu^{2+} (***S***)	38.7	54.2	1.3	3.9	1.3	0.6
	41.5 ± 10.9	62.8 ± 9.3				

Table S2. Detailed experiments in microwave-assisted synthesis of AuNPs**.**

[CBDEB]	T (°C)	t_h (s)	appearance	sample photograph
100 mM	40	300	colorless	n/a
100 mM	60	300	red	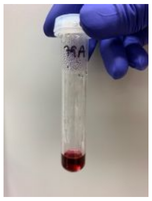
100 mM	60	120	red	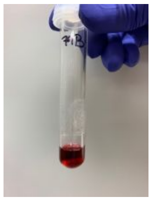
100 mM	60	60	red	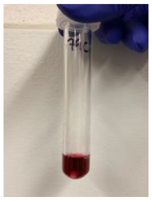
50 mM	60	60	brown	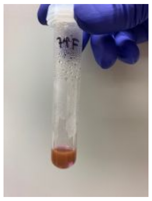
100 mM	60	30	red	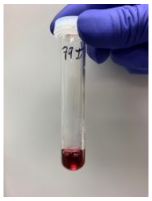
100 mM	60	15	colorless, then red after several min	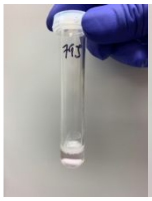 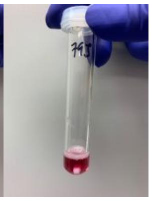

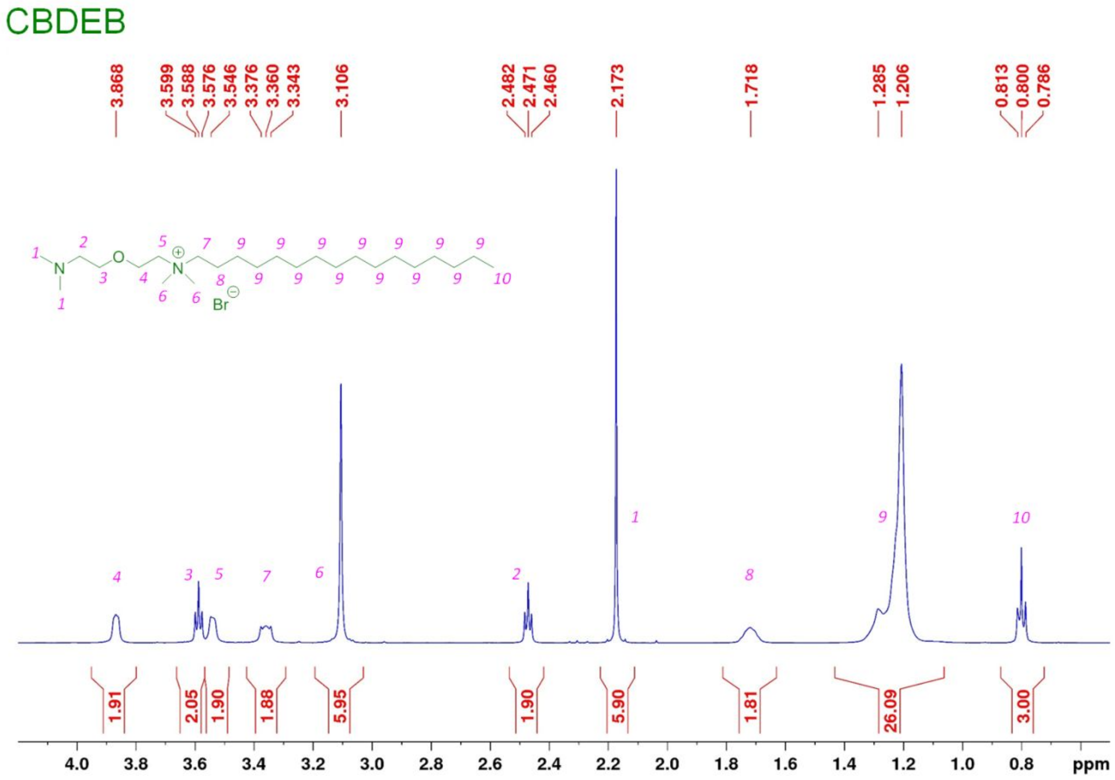

Figure S1. ^{1}H-NMR spectrum of *N*-cetyl-bis(2-dimethylaminoethyl)ether bromide (CBDEB). ^{1}H-NMR (500 MHz, D_2O, [ppm]) δ = 3.86 (s, 2H), 3.59–3.57 (t, 2H, J = 6.0 Hz), 3.54 (s, 2H), 3.37–3.34 (t, 2H, J = 8.0 Hz), 3.10 (s, 6H), 2.48–2.46 (t, 2H, J = 5.5 Hz), 2.17 (s, 6H), 1.71 (s, 2H), 1.20 (s, 26H), 0.81–0.78 (t, 3H, J = 7.0 Hz).

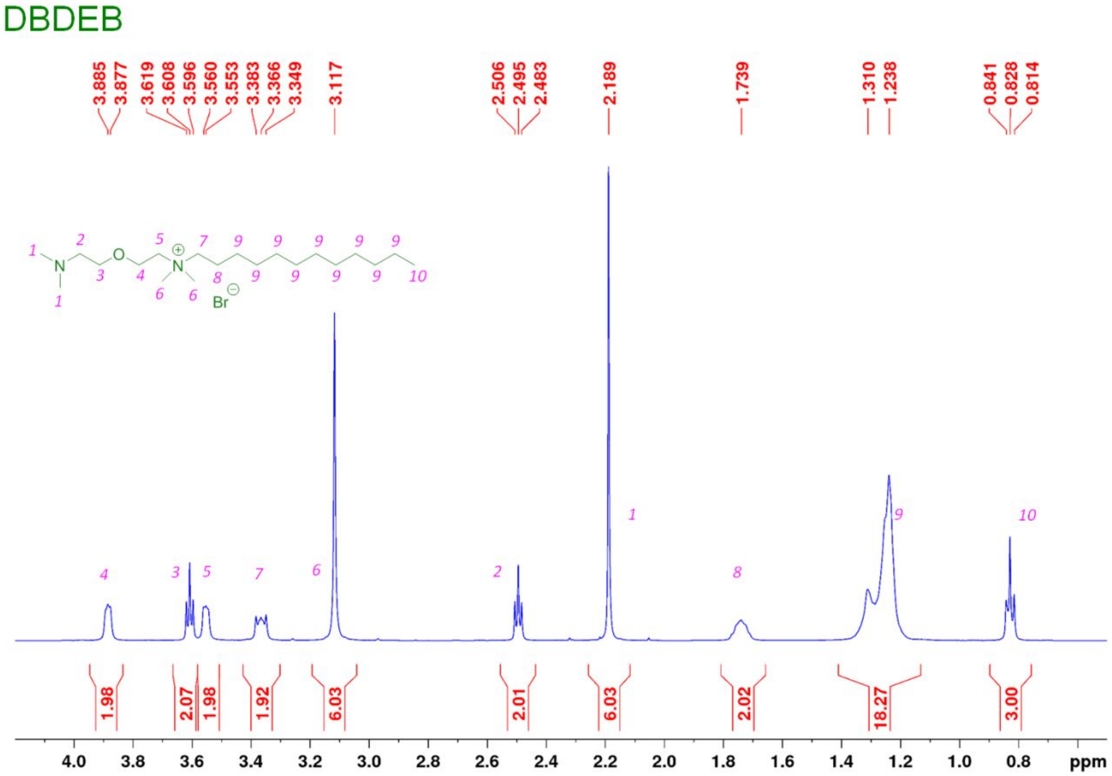

Figure S2. ^{1}H-NMR spectrum of *N*-dodecyl-bis(2-dimethylaminoethyl)ether bromide (DBDEB). ^{1}H-NMR (500 MHz, D_2O, [ppm]) δ = 3.88 (d, 2H), 3.61–3.59 (t, 2H, J = 5.5 Hz), 3.56–3.55 (d, 2H), 3.38–3.34 (t, 2H, J = 8.5 Hz), 3.11 (s, 6H), 2.50–2.48 (t, 2H, J = 5.5 Hz), 2.18 (s, 6H), 1.73 (s, 2H), 1.23 (s, 18H), 0.84–0.81 (t, 3H, J = 6.5 Hz).

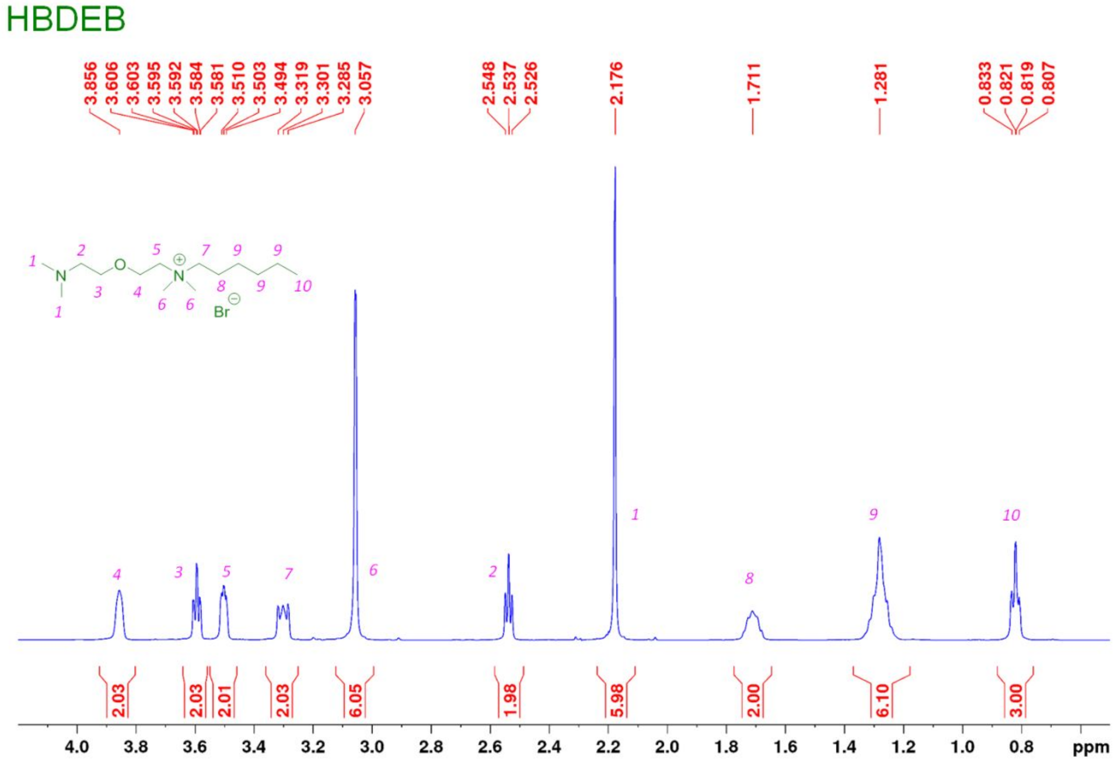

Figure S3. ^{1}H-NMR spectrum of *N*-hexyl-bis(2-dimethylaminoethyl)ether bromide (HBDEB). ^{1}H-NMR (500 MHz, D_2O, [ppm]) δ = 3.85 (s, 2H), 3.60–3.58 (m, 2H), 3.51–3.49 (t, 2H), 3.31–3.28 (t, 2H, J = 9 Hz), 3.05 (s, 6H), 2.54–2.52 (t, 2H, J = 5.5 Hz), 2.17 (s, 6H), 1.71 (s, 2H), 1.28 (s, 6H), 0.83–0.80 (m, 3H).

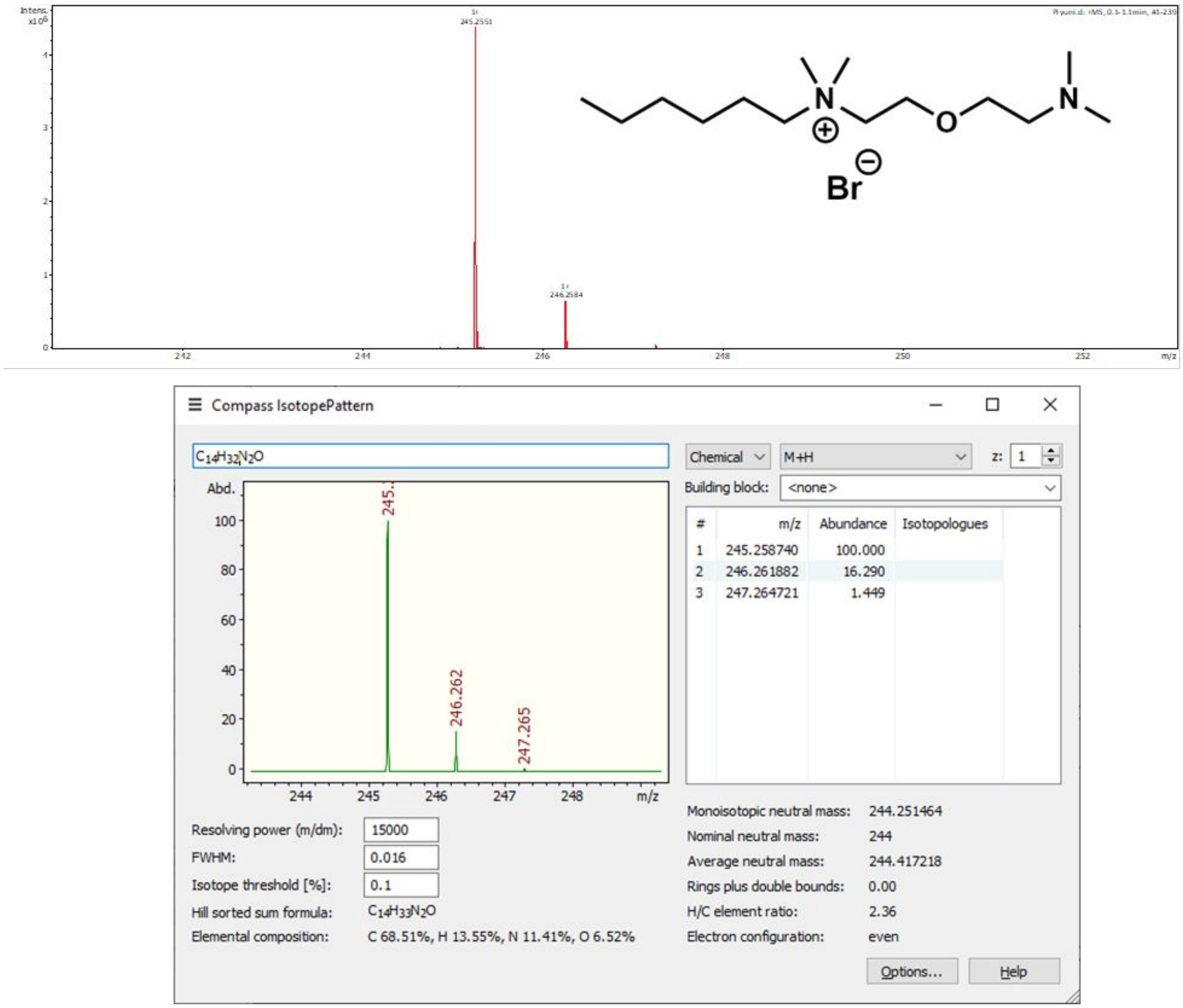

Figure S4. Mass spectrum for HBDEB acquired in positive-ion QTOF-MS mode. The primary ion monoisotopic peak at *m/z* 245.2551 corresponds to the surfactant cation (calculated mass: 245.2587), whereas the lower abundance peaks at *m/z* 246.2584 and ~247.26 are the corresponding carbon-13 isotopic peaks.

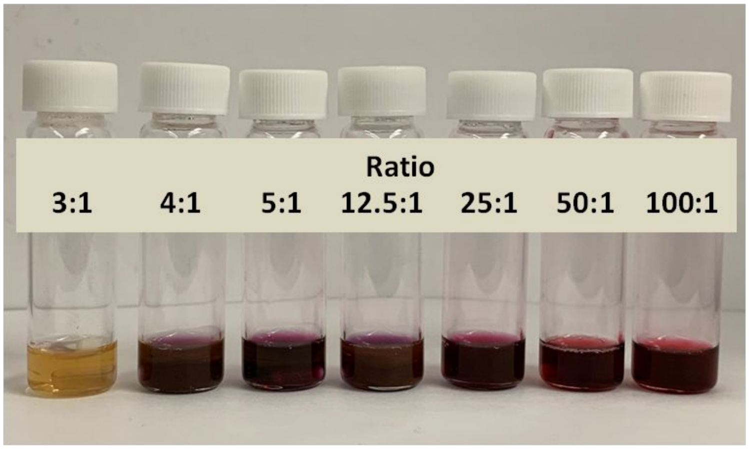

Figure S5. Photograph of AuNP colloids synthesized using various molar ratios of CBDEB surfactant to Au^{3+}; from left to right: 3:1, 4:1, 5:1, 12.5:1, 25:1, 50:1, and 100:1.

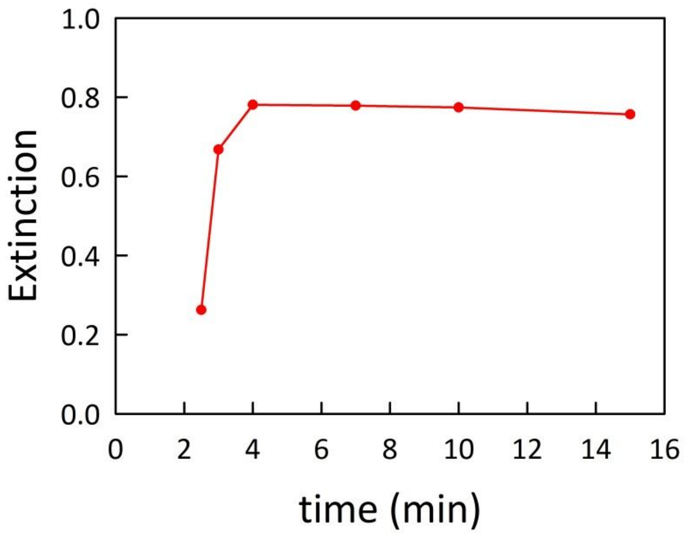

Figure S6. Extinction of AuNPs *vs* time profile. A 20 μL aliquot of 100 mM aqueous $HAuCl_4$ was added to a two-dram vial containing 1.00 mL of 100 μL aqueous CBDEB surfactant solution (50:1 molar ratio surfactant to Au), the sample was immediately put in a reaction block pre-heated at 80 °C and stirred. Aliquots were retrieved from this stock at set time intervals, and the plateau in solution extinction after 4 min suggests an optimal heating/mixing time of 4 min for AuNP synthesis by CBDEB.

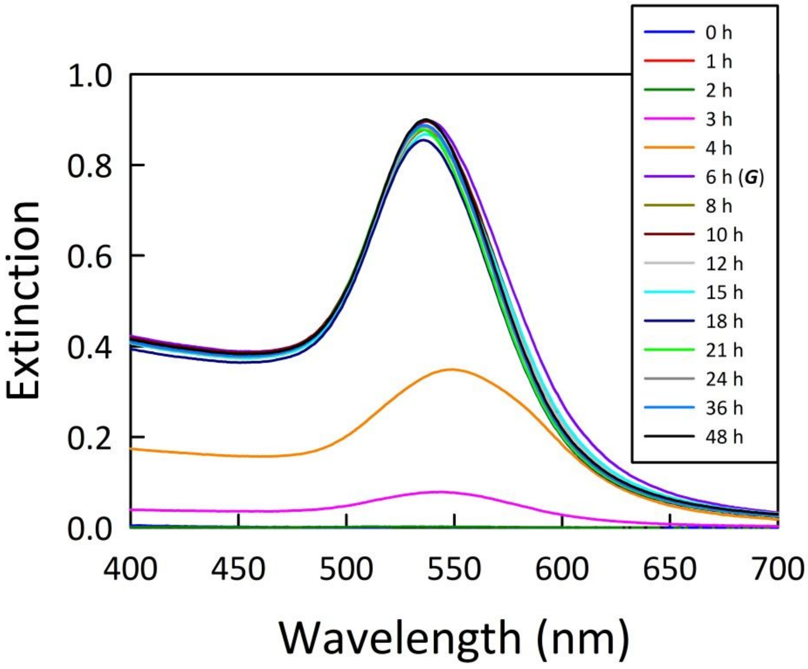

Figure S7. UV−vis spectra depicting the evolution of AuNPs by CBDEB (50:1 molar ratio) stirred at room temperature via the extinction profile of the resulting colloid. Note that the 0, 1, and 2 h plots are all encompassed within the line for the x-axis (i.e., at an extinction of 0) and represent no observable production of AuNPs. Reduction appears to be complete between 4 and 6 h, with only minor narrowing and small extinction shifts occurring after 8 h.

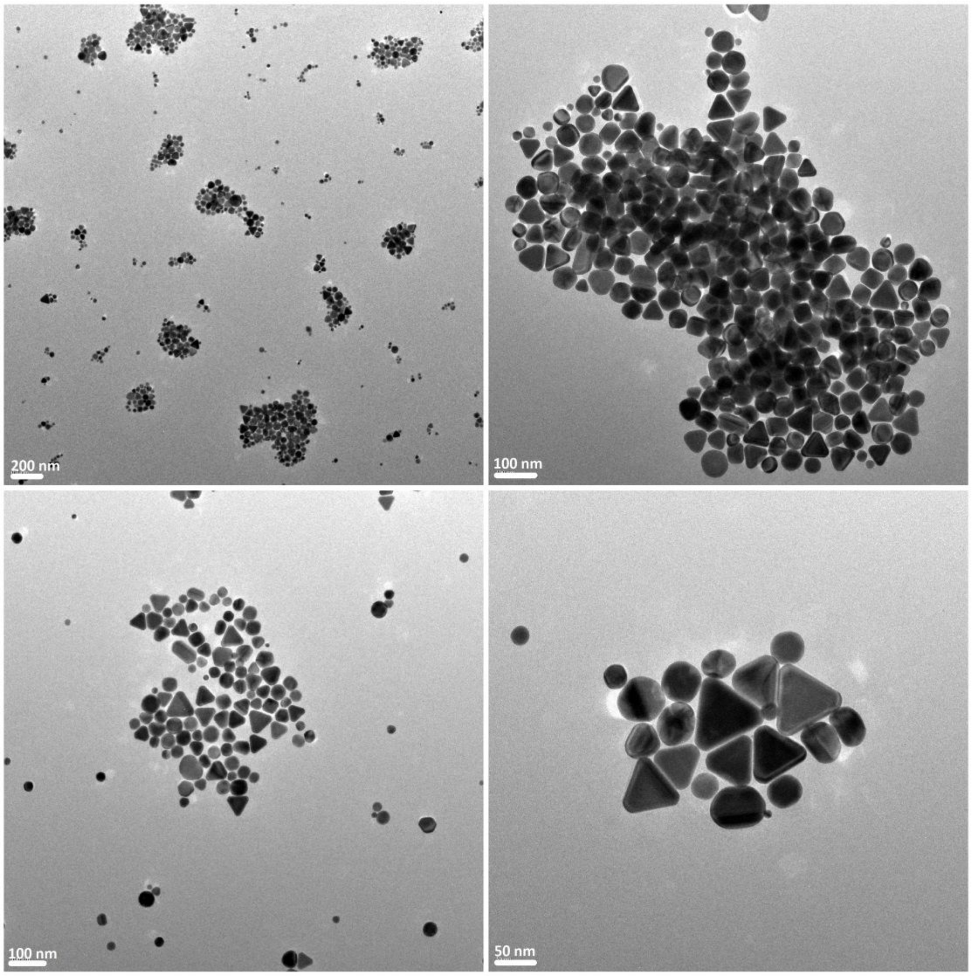

Figure S8. Representative TEM images of AuNPs synthesized at room temperature using CBDEB and a mixing time of 6 h without heating (sample ***G***).

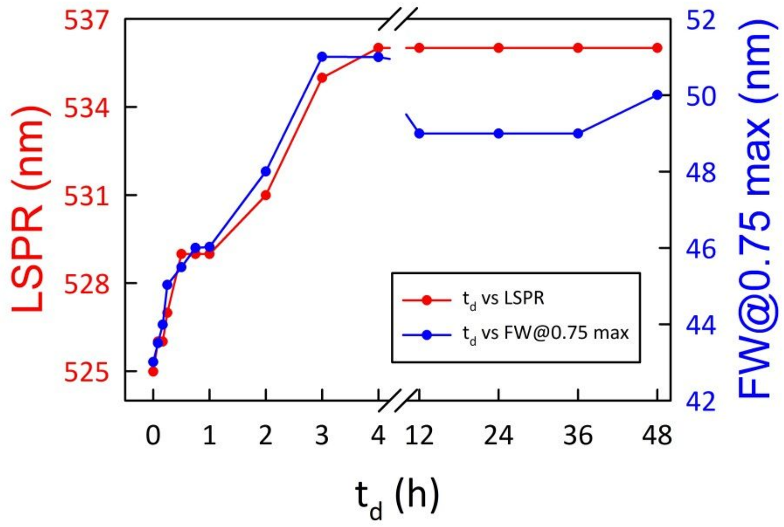

Figure S9. Plot depicting the LSPR maximum and FW@0.75 max extracted from UV−vis spectra of CBDEB-reduced AuNPs colloids produced using a delayed heating method. Here, t_d represents the time during which an aqueous solution comprising a 50:1 molar ratio of CBDEB:Au^{3+} was stirring at room temperature, after which the solution vial was placed in a pre-heated block at 80 °C for reduction. Reduction at 80 °C was concluded after 4 min, and the solution was retrieved and cooled naturally to room temperature before UV–measurements.

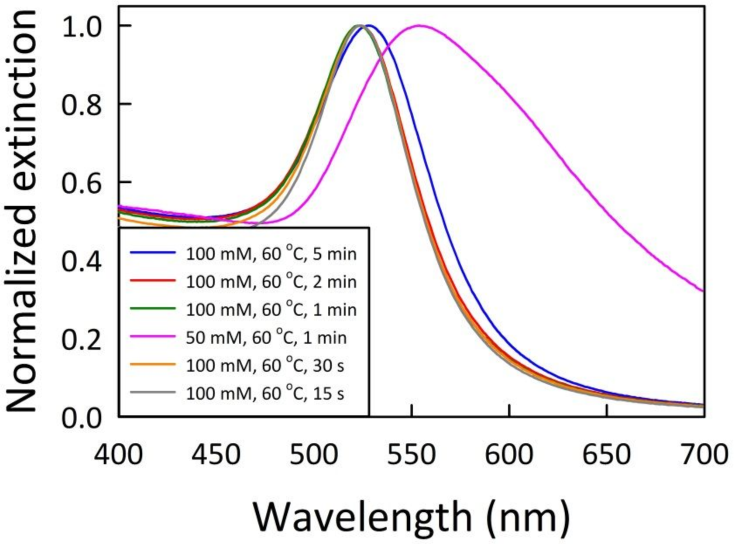

Figure S10. Normalized UV−vis spectra of CBDEB-reduced AuNPs synthesized using microwave irradiation at different conditions. The concentrations indicated in the legend correspond to the [CBDEB], with 100 mM representing a 50:1 molar ratio CBDEB:Au.

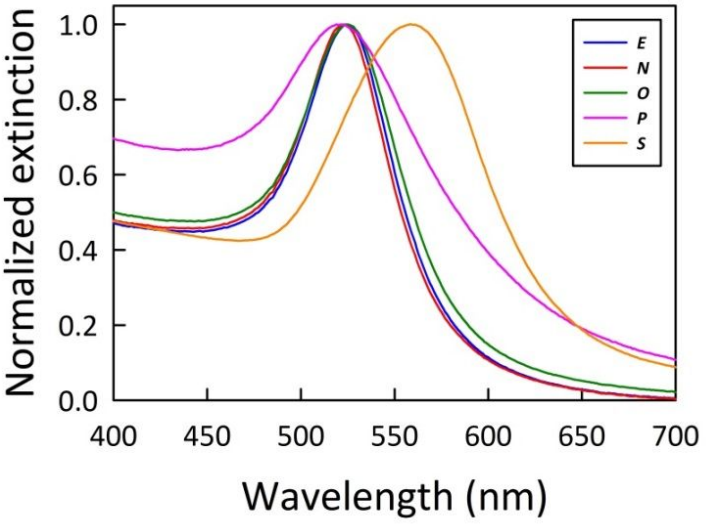

Figure S11. Normalized UV−vis spectra of AuNPs synthesized at various conditions: ***E*** (blue) 50:1 molar ratio of CBDEB:Au using conventional heating (10 min at 80 °C); ***N*** (red) 50:1 molar ratio CBDEB:Au using microwave irradiation; ***O*** (green) synthesized using the shorter chain length DBDEB surfactant; ***P*** (pink) synthesized under alkaline solution; ***S*** (orange) synthesized in the presence of 10 mol% Cu^{2+}, compared with UV−vis band of the AuNPs synthesized at the optimized condition using a conventional heating method (***E***, blue).

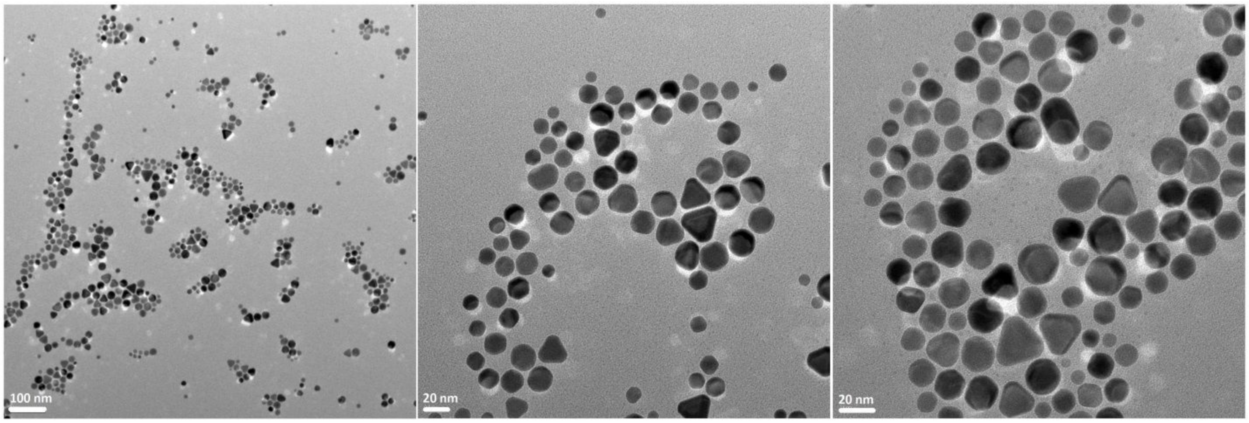

Figure S12. Representative TEM images of AuNPs (sample ***N***) synthesized using microwave irradiation at the optimal conditions (50:1 molar ratio CBDEB:Au, heating at 60 °C, t_h of 30 s).

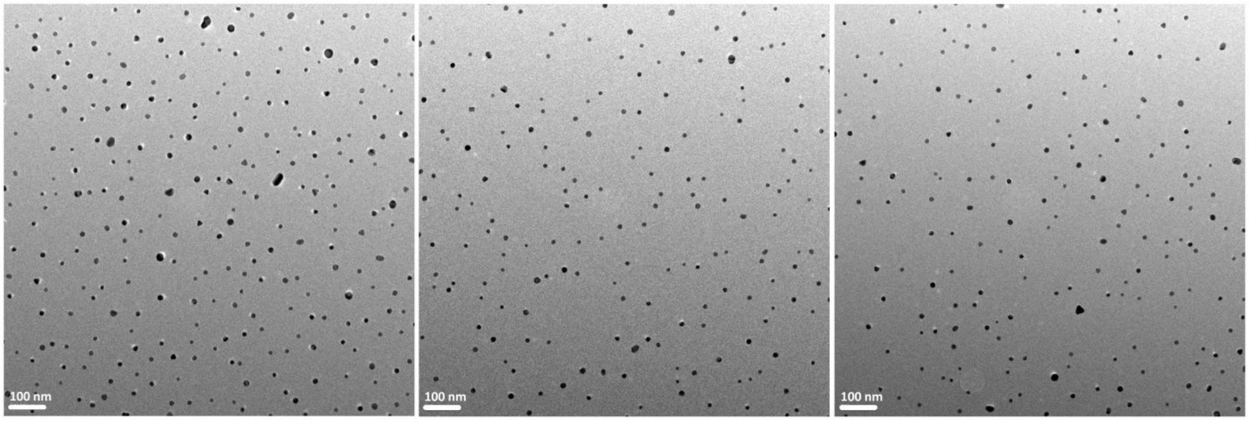

Figure S13. Representative TEM images of AuNPs (sample ***O***) synthesized using DBDEB at a 50:1 molar ratio of DBDEB:Au, with 4 min of heating at 80 °C on a heating block.

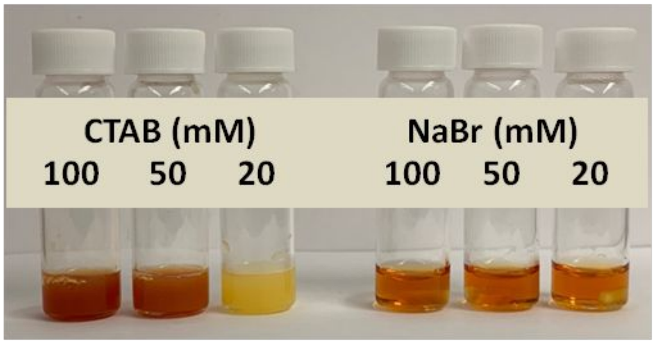

Figure S14. Photograph depicting control studies where our tertiary amine-containing surfactant is replaced with CTAB (left) or NaBr (right) at various concentrations. Addition of 20 μL of 100 mM aqueous $HAuCl_4$ to 80 °C pre-heated solutions of CTAB and NaBr produced yellow to orange solutions, indicating that no AuNP formation occurred.

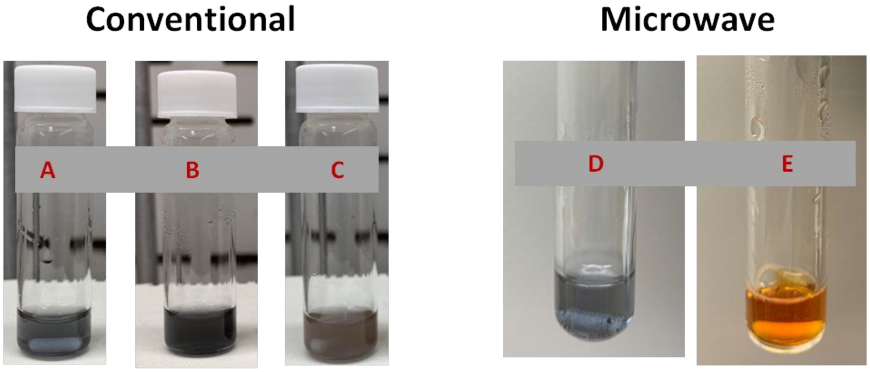

Figure S15. Photographs of control reactions where AuNPs are formed by heating using conventional (left) and microwave (right) methods. Vials (A) and (B) in the left photograph represent solutions comprising a 50:1 molar ratio of DMAEE to Au, where the Au^{3+} solution was added to a conventionally heated DMAEE solution maintained at 60 or 80 °C, respectively, for a 2-min reaction time. Vial (C) represents a similar 80 °C reaction, though the aqueous Au^{3+} solution was added to 1 mL of neat DMAEE. All three products turned dark blue within seconds, indicating larger particles or aggregates. Vials (D) and (E) in the right photograph represent solutions comprising a 50:1 molar ratio of DMAEE or CTAB, respectively, to Au, heated by the microwave method at 60 °C for 30 s. The DMAEE colloid turned blue, indicating aggregation. The CTAB solution remained orange and did not result in AuNPs, and a second solution heated at 80 °C for 5 min also failed to produce AuNPs.

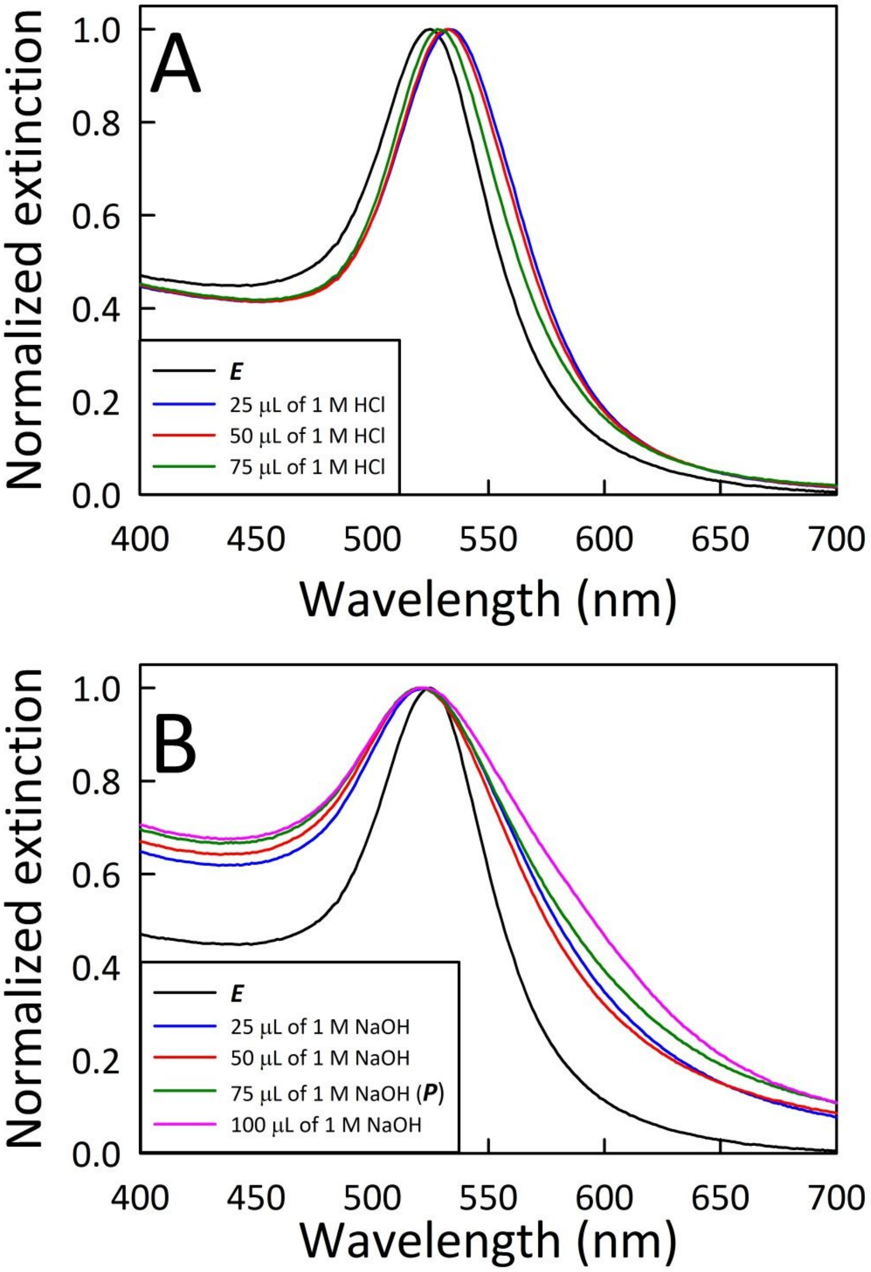

Figure S16. Normalized UV−vis spectra of AuNPs synthesized in (A) acidic conditions and (B) basic conditions. Notably, the solution with 75 μL of added 100 mM HCl failed to produce AuNPs, even after 15 min of heating at 80 °C.

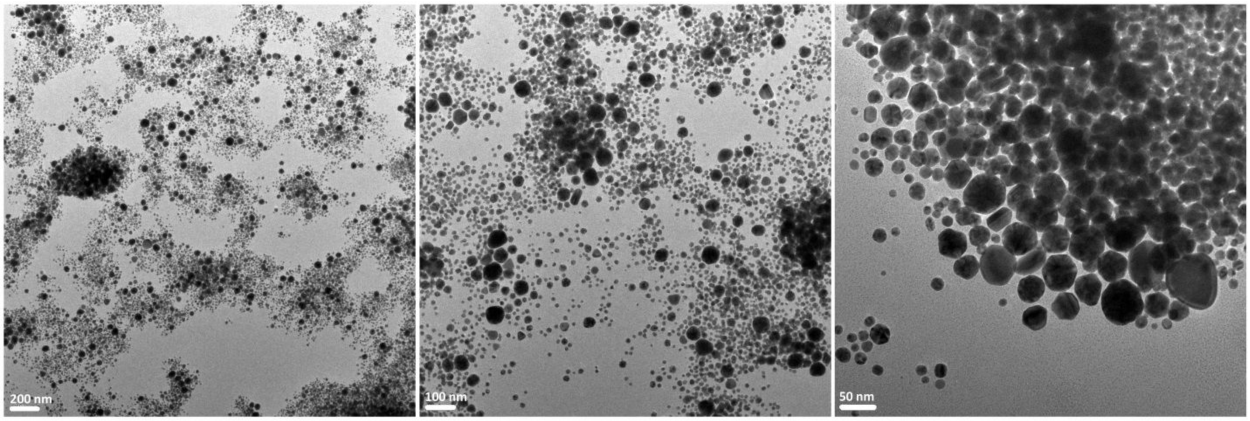

Figure S17. Representative TEM images of AuNPs (sample ***P***) synthesized in the presence of 75 μL of 1 M NaOH, resulting in highly agglomerated particles with large particle size and shape distribution.

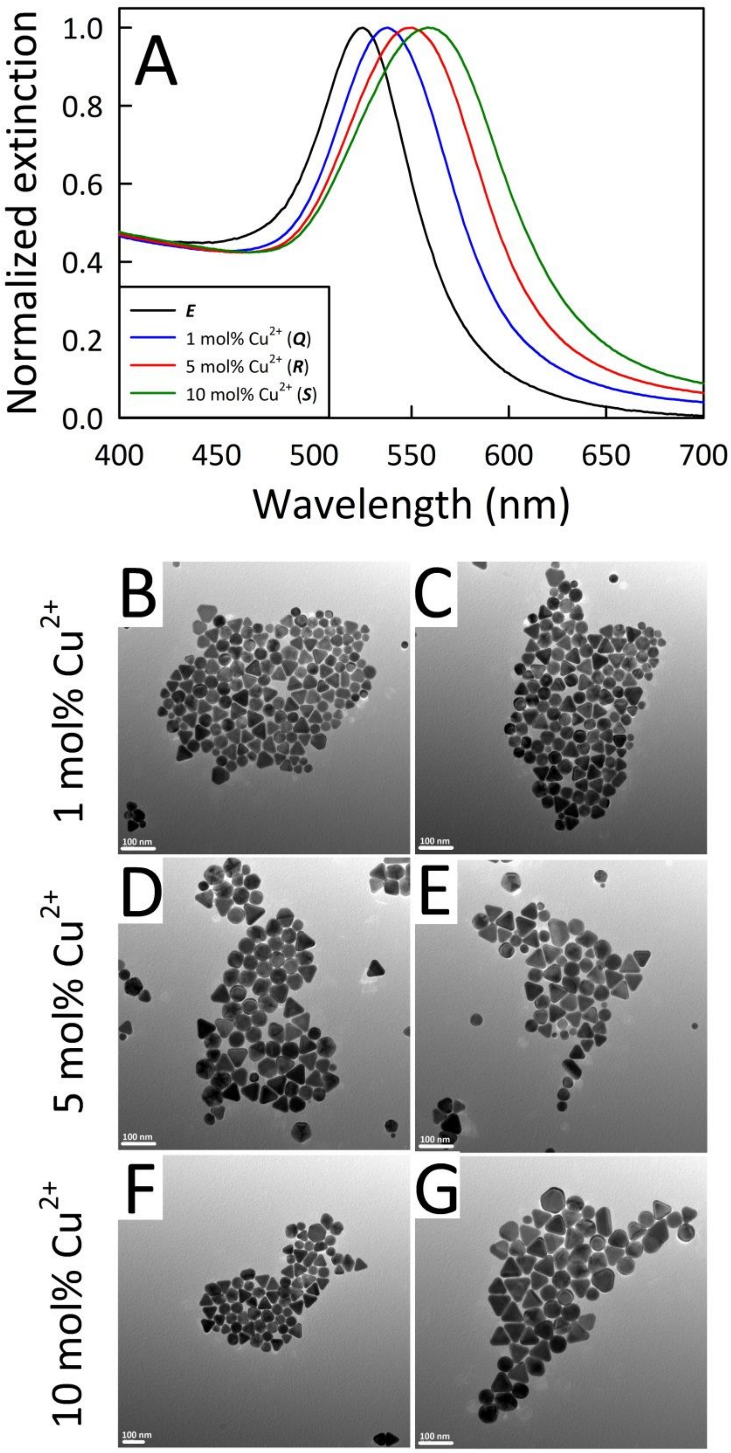

Figure S18. (A) Normalized UV−vis spectra of AuNPs showing LSPR bands bathochromically shifted with increasing $CuSO_4$ amount added; 1 mol% (***Q***, blue), 5 mol% (***R***, red), 1 mol% (***S***, green), compared with AuNP sample synthesized in the optimized condition in the absence of $CuSO_4$ (***E***, black). (B−G) Representative TEM images of samples ***Q*** (B−C), ***R*** (D−E), and ***S*** (F−G).
